# Induction of revertant fibres in the mdx mouse using antisense oligonucleotides

**DOI:** 10.1186/1479-0556-4-3

**Published:** 2006-05-24

**Authors:** Abbie M Fall, Russell Johnsen, Kaite Honeyman, Pat Iversen, Susan Fletcher, Stephen D Wilton

**Affiliations:** 1 Experimental Molecular Medicine Group, Centre for Neuromuscular and Neurological Disorders, University of Western Australia, Nedlands, Perth, 6009, Western Australia; 2 AVI BioPharma, Corvallis, Oregon, USA

## Abstract

**Background:**

Duchenne muscular dystrophy is a fatal genetic disorder caused by dystrophin gene mutations that result in premature termination of translation and the absence of functional protein. Despite the primary dystrophin gene lesion, immunostaining studies have shown that at least 50% of DMD patients, *mdx *mice and a canine model of DMD have rare dystrophin-positive or 'revertant' fibres. Fine epitope mapping has shown that the majority of transcripts responsible for revertant fibres exclude multiple exons, one of which includes the dystrophin mutation.

**Methods:**

The *mdx *mouse model of muscular dystrophy has a nonsense mutation in exon 23 of the dystrophin gene. We have shown that antisense oligonucleotides (AOs) can induce the removal of this exon, resulting in an in-frame mRNA transcript encoding a shortened but functional dystrophin protein. To emulate one exonic combination associated with revertant fibres, we target multiple exons for removal by the application of a group of AOs combined as a "cocktail".

**Results:**

Exons 19–25 were consistently excluded from the dystrophin gene transcript using a cocktail of AOs. This corresponds to an alternatively processed gene transcript that has been sporadically detected in untreated dystrophic mouse muscle, and is presumed to give rise to a revertant dystrophin isoform. The transcript and the resultant correctly localised smaller protein were confirmed by RT-PCR, immunohistochemistry and western blot analysis.

**Conclusion:**

This work demonstrates the feasibility of AO cocktails to by-pass dystrophin mutation hotspots through multi-exon skipping. Multi-exon skipping could be important in expediting an exon skipping therapy to treat DMD, so that the same AO formulations may be applied to several different mutations within particular domains of the dystrophin gene.

## Background

Duchenne muscular dystrophy (DMD), the most well-known of the nine major types of muscular dystrophy, is a severe muscle-wasting disease that arises from mutations in the dystrophin gene (Xp21.2) (review [1,2]). Dystrophin provides structural support to the muscle fibre, and without this protein and its associated protein complex, cell membrane stability becomes compromised, leading to degeneration of the muscle fibres [3]. The significance of dystrophin is demonstrated by the acute pathology resulting from the absence of a functional protein [4,5].

Immunohistochemical analysis of sections of dystrophic muscle using anti-dystrophin antibodies reveals single or clusters of dystrophin-positive fibres called revertant fibres [6,7]. Revertant fibres were observed in at least 50% of DMD patients [8,9], with the incidence in patients' muscles ranging from 0–70% [8-11] and typically less than 1% of muscle fibres in the *mdx *mouse [6,12]. Revertant fibres tend to increase in frequency with age [6,13] in both human and animal models of DMD, possibly indicating a selective advantage over dystrophin negative fibres.

Revertant fibres, observed in DMD patients [9], *mdx *mouse [6] and Golden Retriever Muscular Dystrophy (GRMD) muscle [14], arise from some naturally occurring mechanism where the splicing machinery has been redirected to by-pass the disease-causing mutation and a variable number of flanking exons. These revertant fibres do not elicit an immune response and therefore represent potential templates for functional dystrophins. Studies by Lu *et al *[15] indicated that although a variety of exon skipping combinations were involved in by-passing the *mdx *nonsense mutation, the gene appeared structurally intact in the majority of revertant fibres. Antibody epitope mapping revealed that the loss of 20 exons or more was found in >65% of revertant fibres [15]. Two of the shorter more commonly encountered transcripts detected by RT-PCR arose from the splicing of exon 18 to 35 and exon 13 to 48 [15].

The *mdx *mouse has a nonsense mutation in exon 23 of the dystrophin gene [16] and has been used as an animal model of dystrophin mutations and muscular dystrophy. This model has, and continues to improve our understanding of both the normal function of dystrophin and the dystrophinopathy, as well as aiding in the development of potential therapies. An alternative to replacing or repairing the faulty dystrophin gene is to modify its expression by applying antisense oligonucleotides (AOs) to alter pre-mRNA splicing [17-19] in order to produce a functional protein. In recent years, AOs have been shown to be an effective tool to alter dystrophin pre-mRNA splicing in *mdx *mouse [18-21] and human cell lines [22].

Rather than redirecting splicing by targeting one or two exons to by-pass each specific dystrophin mutation, it may be more effective to induce multiple exon skipping to emulate the mechanism resulting in revertant fibres. Elimination of several exons and introns from the pre-messenger RNA would also enable one cocktail of AOs to treat a variety of mutations clustered within a target region. Furthermore, on the hypothesis that naturally occurring revertant fibres have some selective survival advantage over the neighbouring dystrophic cells and do not appear to induce an immune response, the naturally occurring revertant dystrophin protein may be more functional than that produced by the exclusion of only one exon.

In this report we describe the *in vitro *evaluation and optimisation of an AO cocktail designed to remove exons 19–25, first using 2'-O-methyl phosphorothioate (2OMe) AOs and subsequently phosphorodiamidite morpholino oligonucleotides (PMOs), to induce multiple dystrophin exon skipping in the *mdx *mouse.

## Methods

### Animals

All procedures were approved by the Animal Experimentation Ethics Committee (Approval ID 4/100/373). Normal control C57BL/10ScSn (C57) mice and mutant C57BL/10ScSn-(*Dmd*^*mdx*^) (*mdx*) mice were housed in cages, in temperature controlled rooms (22°C) with a humidity of 50% and a 12:12 hr light-dark cycle. Mice were obtained from the Animal Resources Centre (ARC), Murdoch, Western Australia.

### Amplification of alternatively processed dystrophin gene transcripts

Superscript III one-tube RT-PCR was used with ~50 ng of total RNA as template, in a 12.5 μl reaction using the outer primer sets. 1 μl of RT-PCR solution was used in a secondary nested PCR using AmpliTaq Gold (Applied Biosystems Inc, California). Nested PCR was used throughout and primer sequences are shown in Table 1.

**Table 1:** Primer sequences used for nested PCR analysis

Primer set No.		PCR Primer	Sequence (5'-3')	Full length product (bp)
1	Outer	Exon:13F Exon:27R	GCT TCA AGA AGA TCT AGA ACA GGA GC CTA TTT ACA GTA TCA GTA AGG	
	Inner	Exon 18F Exon 26R	GAA GCT GTA TTA CAG AGT TCT G CCT GCC TTT AAG GCT TCC TT	1250 bp
2	Outer	Exon:13F Exon:27R	GCT TCA AGA AGA TCT AGA ACA GGA GC CTA TTT ACA GTA TCA GTA AGG	
	Inner	Exon 18F Exon 26 R	GAT ATA ACT GAA CTT CAC AG TTC TTC AGC TTG TGT CAT CC	1357 bp
3	Outer	Exon:13F Exon:35R	GCT TCA AGA AGA TCT AGA ACA GGA GC GGT GAC AGC TAT CCA GTT ACT GTT	
	Inner	Exon 13F Exon 35R	CTC GCT CAC TCA CAT GGT AGT AGT G GCC CAA CAC CAT TTT CAA AGA CTC	3406 bp
4	Outer	Exon:13F Exon:50R	GCT TCA AGA AGA TCT AGA ACA GGA GC CCA GTA GTG CTC AGT CCA GGG	
	Inner	Exon 13F Exon 50R	CTC GCT CAC TCA CAT GGT AGT AGT G GGT TTA CAG CCT CCC ACT CAG	5720 bp

### Design and synthesis of antisense oligonucleotides

2'-O-methyl phosphorothioate AOs were prepared on an Expedite 8909 Nucleic Acid Synthesizer (Applied Biosystems Inc) as described previously [23]. The PMOs were supplied by AVI BioPharma (Corvallis, Oregon). To facilitate a direct comparison between the PMO and 2OMe chemistries, both AO chemistries were of the same sequence with nomenclature based on that previously described [19]. Details of AO sequences are shown in Table 2.

**Table 2:** Antisense oligonucleotides used to induce the targeted skipping of murine dystrophin exons 19–25. The optimised cocktail consisting of nine individual AOs (No. 1–9) was used in all experiments to induce exon 19–25 removal. Skipping of single exons resulted in either in-frame (IF) or out-of-frame (OF) transcripts.

No.	Nomenclature	Antisense sequence (5'-3')	Length (bp)	G/C%	No. of AO evaluated	Transcript
1	M19A(+35+65)	GCC UGA GCU GAU CUG CUG GCA UCU UGC AGU U	31	52	12	OF
2	M20A(+23+47)	GUU CAG UUG UUC UGA AGC UUG UCU G	25	44	5	OF
3	M20A(+140+164)	AGU AGU UGU CAU CUG UUC CAA UUG U	25	36		OF
4	M21D(+04-16)	AAG UGU UUU UAC UUA CUU GU	20	25	1	OF
5	M22D(+08-12)	AUG UCC ACA GAC CUG UAA UU	20	40	1	OF
6	M23D(+07-18)	GGC CAA ACC UCG GCU UAC CUG AAA U	25	52	1	IF
7	M24A(+16+40)	CAA CUU CAG CCA UCC AUU UCU GUA A	25	40	6	IF
8	M24A(+78+102)	GAG CUG UUU UUU CAG GAU UUC AGC A	25	40		IF
9	M25D(+06-14)	UAA ACU AGU CAU ACC UGG CG	20	45	1	IF
10	M23D(+02-18) *	GGC CAA ACC UCG GCU UAC CU	20	60		IF

*M23D(+02-18) has been described previously [18, 21]

### Cell culture and transfection

H-2Kb-tsA58 (H-2K) *mdx *myoblasts [24] were cultured as described previously [18]. Briefly, when 60–80% confluent, H-2K myoblast cultures were treated with trypsin (Life Technologies) and seeded at a density of 2 × 10^4 ^per well into 24 well plates, pre-treated with 50 μg/ml poly-D-lysine (Sigma) and 100 μg/ml Matrigel (Becton Dickinson). Cultures were induced to differentiate into myotubes 24 hours prior to transfection by incubation at 37°C, 5% CO_2 _in DMEM containing 5% horse serum. 2OMe AO cocktails were complexed with Lipofectin (Life Technologies) at the ratio of 2:1 lipofectin:AO and used in a final transfection volume of 500 μl/well of a 24-well plate as per the manufacturer's instructions, except that the solution was not removed after 3 hours. Morpholino cocktails were delivered uncomplexed in normal saline at concentrations specified, in a final transfection volume of 500 μl/well.

### Bandstab and direct DNA sequencing

PCR products of interest were isolated from agarose gel and re-amplified using the bandstab technique described previously [25]. A pipette tip was used to stab the desired band which was visualized on a UV transilluminator after staining in ethidum bromide. This was then used to inoculate a PCR reaction and a further 25 cycles of amplification were performed under identical conditions to the previous secondary PCR, except that the annealing temperature was lowered by 5°C. The re-amplified products were purified using spin columns (MoBio) as per the manufacturer's instructions. Direct sequencing was performed using the prism Big Dye-terminator chemistry (V3.1) and a 377A DNA sequencer (Applied Biosystems Inc).

### Intramuscular AO injection and tissue preparation

Equal amounts of each PMO were combined in normal saline to prepare dosages of 2 and 10 μg per 15 μl injection. One *tibialis anterior *muscle of each *mdx *mouse was injected with 15 μl of the AO preparation, the contra-lateral muscle was injected with an equal volume of saline. Two age groups were treated, 11 days (pups) and 16 weeks (adults) and the animals were sacrificed at 2, 4 and 8 weeks after injection (n = 4). The muscles were removed and frozen in iso-pentane cooled in liquid nitrogen, before being cryosectioned and prepared for RNA, protein and immunofluorescence studies.

### Dystrophin immuno-fluorescence

Dystrophin was detected in 6 μm unfixed cryostat sections using the Novacastra NCL-DYS2 monoclonal antibody that reacts with the C-terminus of dystrophin. Immunofluorescence was performed using the Zenon Alexa Fluor 488 labelling kit (Invitrogen), as per the manufacturer's protocol with minor modifications. The initial fixation step was omitted and the primary antibody was used at a dilution of 1:10 with a molar ratio of 4.5:1 [23]. Sections were viewed with an Olympus IX 70 inverted microscope and the images were captured on an Olympus DP 70 digital camera.

### RNA preparation, RT-PCR analysis and western blotting

RNA was extracted from 2–4 mg of cryosections from frozen tissue blocks, using Trizol (Invitrogen) according to the manufacturer's protocol. RT-PCR and secondary amplification were performed across dystrophin exons 18–26, 13–35 and 13–50 using primers detailed in Table 1. Products were fractionated on 2% agarose gels, stained with ethidium bromide and images were captured by a Chemi-Smart 3000 gel documentation system (Vilber Lourmat, Marne La Vallee).

Protein extracts were prepared by adding 120 μl of treatment buffer (125 mM Tris/HCl pH 6.8, 4% SDS, 40% glycerol, 0.5 mM PMSF, 50 mM dithiothreitol, bromophenol blue (Sigma) and protease inhibitor cocktail) per 4 mg *mdx *mouse muscle cryostat sections. Samples were briefly vortexed, sonicated for 2 seconds 4–8 times and heated at 95°C for 5 minutes, before being fractionated at 16°C on a 3–10% SDS gradient gel at pH 8.8 with a 3% stacking gel at pH 6.8. Five or 10 μl of extracts from C57 and 55 μl of extract from treated *mdx *muscle was added to each well. Proteins were transferred from the gel to Hybond nitrocellulose (Amersham Biosciences, Castle Hill) overnight at 18°C, at 290 mA. Dystrophin was visualised using NCL-DYS2 monoclonal anti-dystrophin (Novacastra, Newcastle-upon-Tyne, UK) at a dilution of 1:100 for 2 hours at room temperature, with subsequent detection using the Western Breeze protein detection kit (Invitrogen). Images were captured by a Chemi-Smart 3000 gel documentation system using Chemi-Capt software for image acquisition and Bio-1D software for image analysis (Vilber Lourmat) [23]. One *mdx *muscle extract was mixed with 10 μl of C57 protein to allow normal dystrophin detection in the presence of protein from the *mdx *mouse.

## Results

### Naturally occurring revertant fibre transcripts

**Figure 1:**
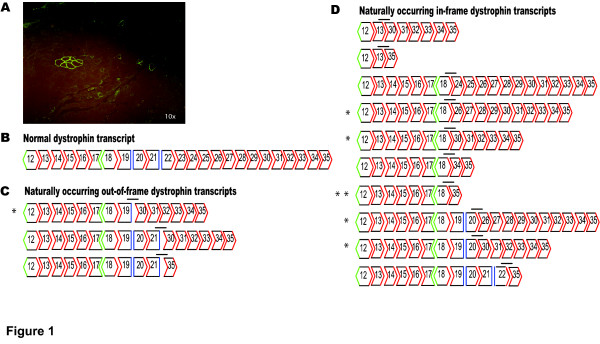
**Dystrophin revertant fibres and transcripts in *mdx *mouse muscle**. (A) A cluster of revertant fibres surrounded by a few single positive fibres in the *mdx *mouse. Tissue was immunostained for dystrophin using NCL-Dys 2. (B-D)Nested RT-PCR was carried out using Primer set 3 inner (Table 1) to study alternative splicing arrangements between exons 13–35. The bar (-) indicates the novel junctions in these transcripts. Unmatched boundary colours identify out-of-frame transcripts. The identity of all transcripts was confirmed by direct sequencing. (B)-Normal dystrophin, (C)-out-of-frame dystrophin transcripts and (D)-in-frame dystrophin transcripts. *Previously reported revertant transcripts *[26] and **[15].

The occurrence of revertant fibres is inconsistent and rare and occurs in dystrophic tissue as either single fibres or small clusters of fibres, observed after immunohistochemical analysis of untreated *mdx *muscle sections (Figure 1A). The diameter of muscle fibres in *mdx *skeletal muscle is less uniform than those in normal (C57BLl/10ScSn) muscle (data not shown). Figure 1B–D indicates the exonic combinations representing alternatively processed dystrophin transcripts detected in *mdx *and normal mouse muscle after RT-PCR amplification of exons 13–35 (Table 1 primer set 3). Over 100 *in vitro *and *in vivo *samples were subjected to this RT-PCR assay, with only thirteen different alternatively spliced transcripts identified, and all but 3 representing in-frame mRNAs. Six of the thirteen transcripts found, indicated by an asterisk in Figure 1A, have been reported previously [15,26].

### Development of 2OMe AOs to induce skipping of exons 19–25

**Figure 2:**
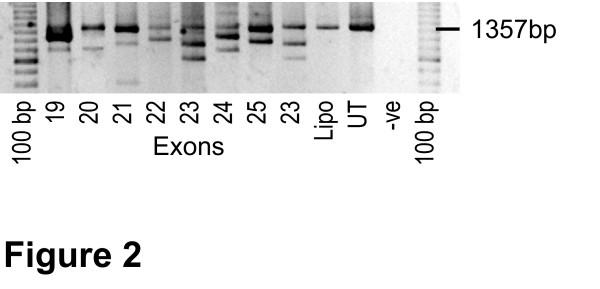
**Exon skipping in cultured *mdx *cells after transfection with AOs directed at targeted exons**. Primer set 2 inner (Table 1) was used for nested PCR amplification to generate the full length product of 1357 bp (indicated). Lane numbers correspond to targeted exons. The induced exon skipping products are 1263 bp (Δ exon 19), 1115 bp (Δ exon 20), 1176 bp (Δ exon 21), 1211 bp (Δ exon 22), 1144 bp (Δ exon 23 A06), 1243 bp (Δ exon 24), 1201 bp (Δ exon 25), (Δ exon19), 1144 bp (Δ exon 23 AO10, Table 2). The 998 bp product corresponds to the removal of exons 22 and 23, a common product of exon 23 targeting.

Despite targeting the obvious donor and acceptor splice sites of individual exons, consistent induction of exon exclusion was not guaranteed. Intra-exonic splicing enhancer (ESE) motifs were targeted and some of these were found to be amenable to redirection of splicing. The likelihood of successful exon skipping after targeting any particular ESE region increased when more than one serine/arginine-rich (SR) binding site was covered by the AO. ESE finder Release 2.0 [27] was used to predict potential binding sites. ESE finder is a human based program, and since it is recognised that there are splicing differences between the human and mouse, the program was used only as a guide. Several AOs were evaluated individually for each exon before selecting the compounds listed in Table 2. All AOs listed in Table 2 induced skipping of the targeted single exon to varying degrees (Figure 2). Exons 20 and 24 were more difficult to remove from the mature mRNA than others, and consistent removal was not achieved with any single AO (data not shown). However, when two apparently ineffective AOs were used in combination, strong and consistent exon 20 and 24 skipping occurred (Figure 2).

**Figure 3:**
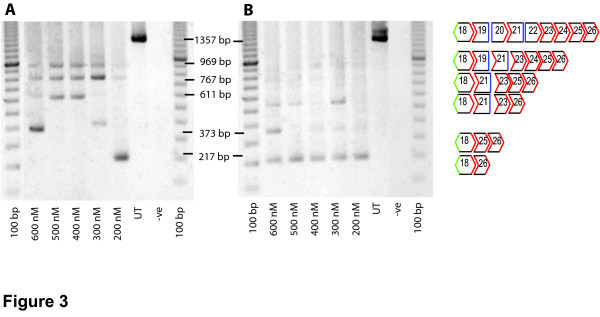
**Induction of multiple exon skipping comparing two different AO cocktails**. The cocktail used in (A) contains M23D(+02–18) and (B) contains M23D(+07–18), other AOs indicated in Table 2. Primer set 2 inner (Table 1) was used for nested PCR amplification where the intact product was 1357 bp long. Both (A) and (B) show the presence of generated multiple bands when the AO cocktail was used *in vitro*. Products were sequenced and transcripts identified. The major induced transcript of 217 bp, corresponds to the deletion of exons 19–25.

AO refinement and optimisation for multiple exon skipping was clearly influenced by the composition of the individual components. Two different AOs targeting exon 23 were evaluated during the optimisation of the 19–25 cocktail. Individually, both AOs induced similarly high levels of exon 23 skipping (Figure 2) but when combined in cocktails, different efficiencies were consistently observed (Figure 3). The inclusion of the 20 mer M23D(+2–18) in the cocktail, did not result in reproducible multiple exon removal (Figure 3a), whereas the inclusion of the 25 mer, M23D(+7–18) in the AO mix, induced consistent skipping over a range of concentrations (Figure 3b). This pattern of exon removal resulting from transfection of the two different AO cocktails was highly reproducible and the AO cocktail containing M23D(+07–18) was used for subsequent studies.

### Evaluation of the 2OMe 19–25 cocktail

**Figure 4:**
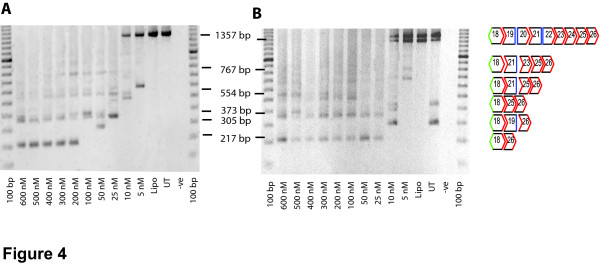
**RT-PCR of shortened transcripts induced in the presence of background alternative splicing**. RT-PCR pattern of exon 19–25 skipping induced in an immortalized culture where there was (A) no evidence of revertant transcripts in the untreated samples, (B) low levels of endogenous alternative splicing visible in the untreated cells. Primer set 2 inner (Table 1) was used for amplification.

Amplification across exons 18 to 26 generated a full length product of 1357 bp that was visible only in the treated samples at AO cocktail transfection concentrations of 5 and 10 nM and in the untreated control (Figure 4). In the majority of the treated samples the full length amplicon was missing. Products representing transcripts missing combinations of other exons were observed and their identity was determined by DNA sequencing (Figure 4).

Titration studies of the 2OMe 19–25 AO cocktail showed consistent induced exon skipping after transfection with a total AO concentration of 200 nM, (approximately 20nM of each AO) (Figure 4a). However, different *mdx *myoblast cultures (both H2K-*mdx *and primary *mdx *myoblasts) demonstrated variable responsiveness to the AO cocktail. Cell densities were kept consistent but exon 19–25 skipping could be induced at lower transfection concentrations in some experiments where revertant transcripts were detected in untreated cells (Figure 4b). Generally, when naturally occurring revertant transcripts were detected in the untreated samples, inducible exon 19–25 skipping was observed after application of an AO cocktail, at concentrations lower than 200 nM. This trend was common to both conditionally immortalised cells and primary *mdx *cells.

The duration of exon skipping after a single *in vitro *AO delivery was assessed. The 2OMe AO cocktail targeting exons 19–25 induced sustained and strong skipping up to 5 days after transfection. However, significant cell death was caused by the transfection reagent (Lipofectin) and results were not consistent after the five day time point (data not shown).

The AO sequences used in the 2OMe cocktail were re-synthesized as PMO compounds to compare the effectiveness of these chemistries. The ratios of individual compounds were the same for both chemistries, however the PMO cocktail was transfected at substantially higher concentration because of the poor uptake of these uncharged compounds *in vitro*. The RT-PCR product representing exon skipping was detectable at low levels (after 7 days) at transfection concentrations above 5 μM (data not shown). Subsequent intramuscular injections of the AO cocktail in the *mdx *mouse were conducted using only the PMO chemistry as we recently reported that PMOs have substantial advantages over the 2OMe chemistry *in vivo *[23].

### *In vivo *studies

**Figure 5:**
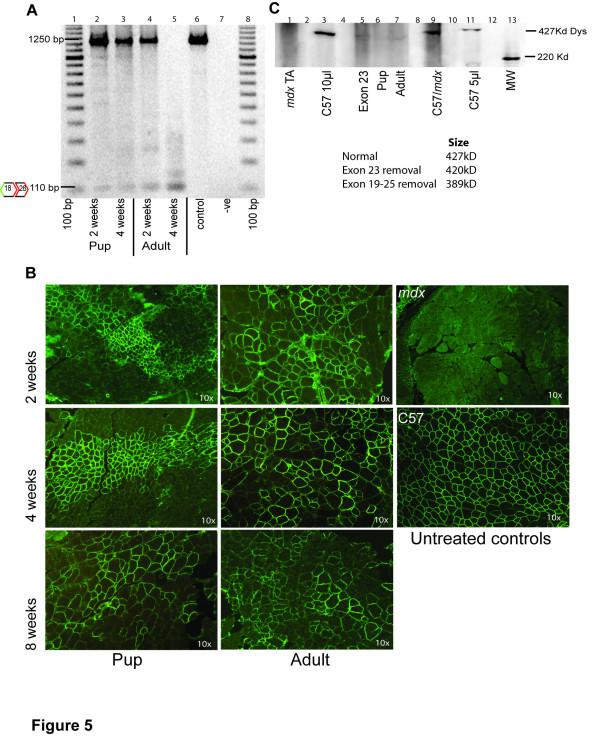
***In vivo *skipping of exons 19–25 induced with a PMO cocktail**. As indicated by the 110 bp product, the PMO cocktail induced the removal of exons 19–25 at 2 and 4 weeks after a single 10 μg intramuscular injection in pups and adult *mdx *mice (A). Primer set 1 inner (Table 1) was used for nested PCR amplification. (B) Dystrophin expression 2, 4 and 8 weeks after intramuscular injection of PMO cocktail in both adults and pups. (C) Western blot analysis was performed one month after injection. A faint protein band of lower than normal dystrophin molecular weight is visible in lanes 6 and 7.

Total RNA, extracted from *mdx *mouse muscle sections (2–3 mg) at 2, 4 and 8 weeks after a single intramuscular injection of 2 or 10 μg of the PMO cocktail, was analysed by nested RT-PCR amplification across dystrophin exons 18–26 (Table 1 primer set 1). Amplification products representing the shortened transcript missing exons 19–25 were observed in all samples from muscles injected with 10 μg of the PMO cocktail. Muscle from mice injected at 11 days or 16 weeks of age contained the shortened transcript at 2 and 4 weeks after a single injection (Figure 5a). A more efficient set of inner primers was used to amplify a full-length transcript of 1250 bp, with the induced transcript represented by a 110 bp product. The expected 110 bp product was not detected in any muscle injected with only the 2 μg dose even though tissue sections had stained positive for dystrophin (data not shown). Immunohistochemical staining with NCL-Dys2 confirmed that the 10 μg injection of the 19–25 PMO cocktail induced widespread dystrophin expression at 2, 4 and 8 weeks after injection in the pups and adult *mdx *mice (Figure 5b). Dystrophin expression appeared maximal at 4 weeks post treatment. Dystrophin staining was localized along the needle track in the sections from the pups, whereas the dystrophin staining in the older *mdx *mice was patchy and more widespread. Dystrophin immunostaining did not appear to differ substantially with the age of the animal. Immunofluorescent staining patterns are similar to those reported with the removal of the single exon 23 [23]. RT-PCR results (Figure 5a) showed the removal of multiple exons with only minor products representing trace amounts of alternatively processed transcripts.

Consistent with the observation that the19–25 transcript was the major induced dystrophin mRNA, western blotting of extracts from treated muscle demonstrated a faint band of induced protein in the samples from pups and adult mice 4 weeks after treatment (Figure 5c). The dystrophin detected in muscles injected with the 19–25 cocktail appeared to be of a lower molecular weight than the C57BL/10ScSn and the exon 23 deleted products. To confirm that the apparent difference in molecular weight was not an artefact of protein loading, 15% normal muscle protein extract was mixed with 85% untreated *mdx *muscle protein in the loading buffer. Levels of protein were too low to be detected at 2 or 8 weeks, even though dystrophin was observed by immunofluorescent staining of muscle sections.

### Induced revertant fibres

**Figure 6:**
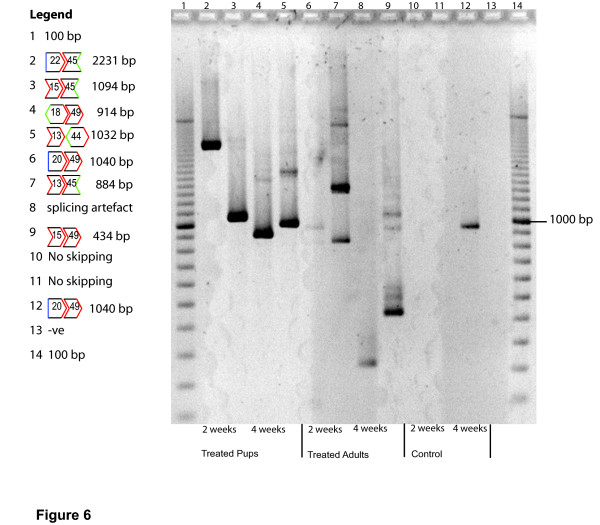
**Amplification of dystrophin exons 13–50, RNA extracted from treated and untreated *mdx *muscle**. Primer set 4 inner (Table 1) was used for nested PCR amplification. The identity of the alternatively spliced products is shown with the reading frame indicated. Lanes 3, 4, 7 and 9 correspond to the samples used in Figure 5a lanes 2–5.

To determine if other multiple skipping events occurred as a consequence of treatment with AO cocktails, long range RT-PCR across exons 13–50 was performed on untreated and treated samples from both pups and adult *mdx *mice at the 2 and 4 week time points (Figure 6). The frequency of revertant transcripts appeared to be higher in PMO cocktail-treated samples than in sham-injected muscle. Only one of the 4 untreated samples contained a naturally occurring in-frame revertant transcript missing exons 20–49, whereas all eight of the treated samples contained additional shorter transcripts. The transcript skipping exons 20–49 was also found in one of the PMO cocktail treated samples, with most of the induced shortened transcripts found to be in-frame (Figure 6). Due to either its size, the quality of the RNA, or the efficiency of the cDNA synthesis and amplification, the full length product of 5720 bp was not generated by this assay and the reaction was biased towards the amplification of shorter alternatively processed products.

## Discussion

A study of BMD dystrophin gene rearrangements that result in an altered but partially functional protein, readily identifies dispensable domains within the dystrophin protein [28]. There are many cases of asymptomatic BMD, where patients have only been diagnosed late in life. England *et al *[29] reported a BMD case identified at 60 years of age, with a deletion of exons 17–48 that encompassed 46% of the gene. The reading frame rule proposed by Monaco *et al *[4,5] holds true for over 90% of dystrophin mutations. However, there are exceptions and it has been reported that in-frame deletions exceeding 36 exons are generally associated with a severe clinical phenotype [30-32]. Nevertheless, other reports of milder, though variable phenotypes with large in-frame deletions, involve the loss of up to 66% of the dystrophin gene [33]. It would appear that most exons encoding the rod domain may be deleted without substantial loss of function. The loss of a single exon that either codes for a crucial binding domain or disrupts the reading frame will have catastrophic consequences on dystrophin function [29,33,34]. AOs must be designed and optimised to remove the exon containing the mutation, and/or surrounding exons, to restore or maintain the reading frame. While DMD and most BMD patients lack full-length dystrophin, expression of shorter dystrophin isoforms (Dp260, Dp140, Dp116 and Dp71) occurs in different muscle and non-muscle tissues, with inter-patient variation depending on the position of the primary lesion in the dystrophin gene [35].

The aim of multiple exon skipping was to induce a previously identified, naturally occurring revertant dystrophin transcript [26]. Revertant fibres arise from spontaneous exon skipping events that occur at low levels in both normal and dystrophic muscle [17]. We focussed on identifying revertant fibre transcripts between exons 13 and 35 (~29% of the dystrophin gene) in the *mdx *mouse model. It has been proposed that the dystrophin in revertant fibres arises from alternatively spliced transcripts that lack both the mutant exon and a variable number of adjacent exons [15,36]. Splicing is a very complex process with many cis and trans elements contributing to the selection of, and efficiency with which splice sites are recognised and exons are joined during pre-mRNA processing. The strength of the 3' and 5' splice sites, branch point sequences, exonic splicing enhancers and silencers, exon length, and secondary structure all play a role in pre-mRNA processing [37-40]. Alternative splicing has now been recognised as a major mechanism for generating protein diversity in higher eukaryotes [41]. Alternative splicing does occur naturally in the dystrophin gene transcript, but the role of many isoforms has yet to be elucidated [42].

It appears that revertant fibres arise from some alternative splicing mechanism, as evidenced by the presence of the shorter dystrophin isoforms, however the low frequency would suggest some error in splicing, where a localised event within fibres rescues dystrophin expression. Although too few in number to be of any therapeutic benefit, the presence and persistence of these dystrophin positive fibres implies that they do not elicit an immune response.

The concept of a localised change in the general splicing machinery or an altered ratio of SR proteins leading to alternative splicing to by-pass the dystrophin gene lesion seems unlikely. It is tempting to speculate that perhaps some novel microRNA is expressed in the revertant fibres, leading to natural exon skipping in a single cell, with clusters of dystrophin positive fibres suggesting a clonal origin. MicroRNAs have been implicated in a variety of cell processes, including apoptosis, translation andsplicing [43,44]. Regardless of the mechanism used to induce revertant dystrophin, it is possible that the addition of AOs is somehow enhancing the production or action of some microRNAs.

Substantial optimisation was performed in assembling the AO cocktail, with a number of AOs evaluated before selecting the combination shown in Table 2. Although there was no common pre-mRNA motif that could be targeted to induce reliable and sustained exon skipping, in general longer AOs were found to be more effective [45]. Mouse dystrophin exon 19 has been studied previously and may be regarded as an easy exon to remove from the dystrophin mRNA [46]. Ten AOs directed at the acceptor, ESE and donor splice sites all induced exon 19 skipping. In contrast exons 20 and 24 proved much harder to displace from the mature mRNA. Five AOs were directed at exon 20, and six at exon 24. Individually, these AOs proved ineffective at inducing skipping of the target exon but the combinations described here were most effective. One could speculate that this indicates some exons have multiple motifs necessary for exon recognition by the splicing machinery and more than one target must be masked to redirect splicing. In these cases it may be necessary to apply multiple AOs to target a single exon for mRNA excision.

The AOs evaluated for exon 23 removal proved to be crucial to the 19–25 cocktail. Individually both AOs, M23D(+02–18) and M23D(+07–18), removed exon 23 but the 25mer was the more effective of the two AOs. It was noted that a single AO could substantially influence the efficiency of the AO cocktail with respect to multiple exon skipping. In order to induce exon 19–25 skipping with the AO cocktail containing M23D(+02–18), it was necessary to adjust the ratios of each AO in the mixture (data not shown). However, if equal molar amounts of all AOs were used then the M23D(+02–18) cocktail did not reliably induce exon 19–25 skipping. Upon inclusion of M23D(+07–18) in the mixture, consistent and reliable multiple exon skipping was induced (Figure 3). Further optimisation of all other AOs in the mixture could be undertaken, but since consistent generation of the desired transcript was achieved, it was decided to undertake subsequent *in vitro *and *in vivo *experiments with the cocktail containing M23D(+07–18).

Administration of the AO cocktail containing M23D(+07–18) appeared to increase the incidence of dystrophin revertant transcripts in *mdx *myogenic cells and tissue. RNA extracted from treated and untreated muscle was subjected to RT-PCR across exons 13–50. Each treated sample had one or more alternatively processed dystrophin gene transcripts, whereas shorter products were rare in untreated samples.

The subtle variations in protein band migration observed on the western blot (Figure 5c) indicates the size differences between dystrophin of normal length (427 kD), exon 23 deleted (420 kD) and the removal of exons 19–25 (389 kD). The concept of multiple transcripts is consistent with observation of "fuzzy" bands on western blots of treated muscle that were fractionated specifically to enhance resolution and size differentiation. Multiple exon skipping events could occur at many positions in the *mdx *dystrophin gene transcript and still by-pass the nonsense mutation.

We have recently shown that PMOs are more effective than 2OMe AOs at inducing exon 23 skipping in the dystrophin gene transcript [23]. PMOs exhibit very low toxicity in treated cells [47] and have been reported to have minimal non-antisense effects [48]. Intramuscular injections of PMOs produced no obvious adverse reactions at or around the injection site (data not shown). RT-PCR, immunohistochemistry and western blot all confirmed that induced skipping of exons 19–25 was far more efficient *in vivo *with PMOs than with 2OMe AOs (data not shown).

Dystrophin was still detectable 8 weeks after a single intramuscular injection of the PMO cocktail into 11 day old pups and 16 week old adult mice, although differences in immunostaining patterns were apparent. The dystrophin staining pattern in the pups appeared strongly localised at the injection site and consistently positive, whereas the pattern in adult mice was more patchy and widespread. This may have been due to the amount of degeneration:regeneration that had occurred in the adult *mdx *mouse, and to the fact that the pups were injected prior to the extensive necrosis that occurs at around 18 days of age [49].

In some cases, the removal of a single exon would not be sufficient to address the disease-causing mutation and a cocktail of two or more AOs would be required to restore the reading frame. For example, the intron 6 splice site mutation in the GRMD canine model of DMD leads to the skipping of exon 7, with a subsequent frame-shift in the dystrophin mRNA [50]. The minimum change to restore the reading frame requires the removal of exons 6 and 8 and was recently reported by McClorey *et al *[51]. Similarly, any nonsense mutation in exons 6, 7 or 8 of the human dystrophin gene would require removal of all 3 exons to by-pass the mutation and still maintain the reading frame. For these reasons, multiple exon skipping and the application of AO cocktails will be an absolute requirement to address some DMD mutations

## Conclusion

The removal of exons 19–25 in the *mdx *mouse provides evidence that multiple exon skipping is feasible and that clusters of mutations in the dystrophin gene could be corrected with a cocktail of AOs. Once a comprehensive set of AOs are designed these could theoretically benefit >75% of all DMD patients. One of the major limitations is to gain regulatory approval for the clinical use of so many different compounds [52,53]. The cost of safety and toxicology testing alone could render exon-skipping a non-viable approach for all amenable dystrophin mutations, in particular, those defects occurring outside the recognised deletion hot-spots. AO cocktails to induce multiple exon skipping could significantly lower the number of preparations required to address clustered dystrophin mutations in different families [53].

## Competing interests

The author(s) declare that they have no competing interests.

## Authors' contributions

AF carried out the molecular genetic studies, immunohistochemistry, participated in its design and coordination and helped to draft the study, RJ carried out the Western Blots, KH helped to draft the study, PI designed and supplied the PMOs, SF and SW conceived the study, participated in its design and coordination and manuscript preparation. All authors read and approved the final manuscript.
